# Genomic insights into the circulation of pandemic fluoroquinolone-resistant extra-intestinal pathogenic *Escherichia coli* ST1193 in Vietnam

**DOI:** 10.1099/mgen.0.000733

**Published:** 2021-12-14

**Authors:** Quynh Nguyen, To Thi Nguyen Nguyen, Phuong Pham, Vinh Chau, Lan Phu Huong Nguyen, Toan Duc Nguyen, Tuyen Thanh Ha, Nhi Thi Quynh Le, Duong Thuy Vu, Stephen Baker, Guy E. Thwaites, Maia A. Rabaa, Duy Thanh Pham

**Affiliations:** ^1^​ Oxford University Clinical Research Unit, Ho Chi Minh City, Vietnam; ^2^​ The Hospital for Tropical Diseases, Ho Chi Minh City, Vietnam; ^3^​ Children’s Hospital 1, Ho Chi Minh City, Vietnam; ^4^​ The University of Medicine and Pharmacy, Ho Chi Minh City, Vietnam; ^5^​ Cambridge Institute of Therapeutic Immunology & Infectious Disease (CITIID), Department of Medicine, University of Cambridge, Cambridge, UK; ^6^​ Centre for Tropical Medicine and Global Health, Nuffield Department of Medicine, University of Oxford, Oxford, UK

**Keywords:** ExPEC, *E. coli*, ST1193, bloodstream infections, fluoroquinolone resistance

## Abstract

Extra-intestinal pathogenic *Escherichia coli* (ExPEC) ST1193, a globally emergent fluoroquinolone-resistant clone, has become an important cause of bloodstream infections (BSIs) associated with significant morbidity and mortality. Previous studies have reported the emergence of fluoroquinolone-resistant ExPEC ST1193 in Vietnam; however, limited data exist regarding the genetic structure, antimicrobial resistance (AMR) determinants and transmission dynamics of this pandemic clone. Here, we performed genomic and phylogenetic analyses of 46 ST1193 isolates obtained from BSIs and healthy individuals in Ho Chi Minh City, Vietnam, to investigate the pathogen population structure, molecular mechanisms of AMR and potential transmission patterns. We further examined the phylogenetic structure of ST1193 isolates in a global context. We found that the endemic *E. coli* ST1193 population was heterogeneous and highly dynamic, largely driven by multiple strain importations. Several well-supported phylogenetic clusters (C1–C6) were identified and associated with distinct *bla*_CTX-M_ variants, including *bla*_CTXM-27_ (C1–C3, C5), *bla*_CTXM-55_ (C4) and *bla*_CTXM-15_ (C6). Most ST1193 isolates were multidrug-resistant and carried an extensive array of AMR genes. ST1193 isolates also exhibited the ability to acquire further resistance while circulating in Vietnam. There were phylogenetic links between ST1193 isolates from BSIs and healthy individuals, suggesting these organisms may both establish long-term colonization in the human intestinal tract and induce infections. Our study uncovers factors shaping the population structure and transmission dynamics of multidrug-resistant ST1193 in Vietnam, and highlights the urgent need for local One Health genomic surveillance to capture new emerging ExPEC clones and to better understand the origins and transmission patterns of these pathogens.

## Data Summary

Metadata associated with ST1193 isolates from our study are included in Table S1 (available in the online version of this article). Accession numbers and associated metadata for ST1193 genomes from the Enterobase are described in Table S2.

**Impact Statement**

Extra-intestinal pathogenic *Escherichia coli* (ExPEC) are common causes of bloodstream infections (BSIs), urinary tract infections and meningitis. The continuing emergence and global dissemination of various ExPEC strains pose public health challenges. These bacteria are often resistant to multiple antibiotics, which limits treatment options and worsens clinical outcomes. Recently, a particular fluoroquinolone-resistant ExPEC, called ST1193, has emerged and spread internationally, although its circulation and antibiotic resistance in low- and middle-income settings are still poorly understood. Here, we decoded the genetics of ST1193 isolates from BSIs and healthy carriers in Ho Chi Minh City, Vietnam, to investigate their genetic relationships, and to understand how these organisms develop antibiotic resistance and spread in the local population. Our study uncovered the endemic circulation of multiple ST1193 clusters associated with distinct *bla*_CTX-M_ variants, including *bla*_CTXM-27_, *bla*_CTXM-55_ and *bla*_CTXM-15_, as a result of importation events from other countries. Most ST1193 isolates were resistant to multiple antimicrobial drugs, and the ST1193 isolates from BSIs and healthy individuals were genetically closely related. Our study provides new understanding of the transmission dynamics and antibiotic resistance of ST1193 isolates in Vietnam, and raises the importance of local/regional cross-sector surveillance to capture newly emerging bacterial pathogens and understand their origins and transmission patterns for better control and prevention.

## Introduction

Bloodstream infection (BSI) is an important global public health concern and a cause of significant morbidity and mortality [1–7]. BSIs affect an estimated 30 million people per year, potentially resulting in 6 million deaths [8]. Globally, about 3 million newborns and 1.2 million children suffer from BSIs annually [9]. In recent decades, extra-intestinal pathogenic *Escherichia coli* (ExPEC) have become a predominant cause of BSIs, and show an increasing resistance to common drugs of choice, including third-generation cephalosporins and fluoroquinolones [10, 11]. The emergence and global spread of extended-spectrum β-lactamase (ESBL)-producing ExPEC is of great concern, as these organisms often display a multidrug-resistant (MDR) phenotype (defined as acquired non-susceptibility to at least one agent in three or more antimicrobial categories [12]), which further limits treatment options. Previous studies have shown that BSIs caused by ESBL-producing ExPEC are commonly associated with worse prognoses and higher mortality rates, largely due to administration of ineffective initial antimicrobial therapy [13–15].

Multi-locus sequence typing (MLST) and genomic analyses of global ExPEC isolates have provided insights into its complex and dynamic population structure, as well as the mechanisms of antimicrobial resistance (AMR) in these organisms. Multiple pandemic ExPEC lineages have been found to co-circulate globally, of which ST131 is the predominant clone, followed by ST393, ST73, ST69 and ST95 [16, 17]. However, the evolutionary processes and mechanisms driving the international spread of these clones are largely unknown. In the last 25 years, a newly emerging ExPEC clone, ST1193, has disseminated across the globe, and is generally associated with BSIs, urinary tract infections and meningitis [18–20]. ST1193 isolates are O type O75, emerge from the clonal complex ST14 (ST14cc) and belong to phylogenetic group B2, which is responsible for most human infections [21]. ST1193 isolates often exhibit MDR plus a fluoroquinolone-resistant phenotype, and have a propensity to gain additional resistance via acquisition of ESBL-encoding plasmids [21]. To date, the majority of ST1193 isolates have been identified from human samples [22] with limited overlap with animals [23], suggesting that human-to-human transmission is likely to be the primary transmission pathway; however, more thorough investigations are needed to identify ST1193’s sources and mechanisms of spread.

In Vietnam, data from hospital-based surveillance in 16 hospitals between 2012 and 2013 showed that *E. coli* was the predominant pathogen identified from blood and cerebrospinal fluid; 50 % of these *E. coli* were resistant to fluoroquinolones and third-generation cephalosporins [24]. However, data on the genetic structures, transmission pathways and AMR profiles of ExPEC clones are lacking. In this study, we performed genomic and phylogenetic analyses of *E. coli* ST1193 isolates recovered from patients with BSIs and human carriers within the same population in Vietnam to delineate this lineage’s population structure, characterize its molecular resistance mechanisms and investigate its potential transmission patterns.

## Methods

### Bacterial isolates

A total of 46 ST1193 isolates (39 from BSI cases and seven from human carriers) were included in this study (Table S1). Isolates from BSIs include: eight isolates from a prospective observational study of neonatal sepsis in Children’s Hospital 1 (CH1) in Ho Chi Minh City (HCMC), Vietnam, between 2017 and 2019 (OxTREC number: 35-16 and CH1 approval number: 73/GCN/BVND1) [25], and 31 isolates from a retrospective study of BSIs in the Hospital for Tropical Diseases (HTD), a tertiary healthcare facility for infectious diseases in HCMC, between 2010 and 2014 (HTD approval number: CS/ND/14/20). The ages of neonates with sepsis ranged from 4 to 13 days at admission (mean gestation age: 39 weeks). All neonatal sepsis cases were late-onset (occurring after 72 h of birth) and the sources of sepsis were unknown. Regarding pre-existing diseases, one case had cogenital heart and gastrointestinal (GI) abnormality, and one had cogenital renal and GI abnormalities. All ST1193 isolates from the BSI study at HTD were identified from adults, with ages ranging from 18 to 81 years (median age: 50 years); there were 22 females and nine males. Isolates from human carriers (collected from rectal swabs/faecal specimens) include five isolates from healthy children enrolled in a cohort study of paediatric diarrhoea in HCMC, conducted between 2014 and 2016 (OxTREC number: 1058-13) [26], and two isolates from *Shigella *-infected children enrolled in a prospective, observational study of children hospitalized for diarrhoea in HCMC between 2014 and 2016 (OxTREC number: 0109) [27].

### Microbiological methods

#### Blood culture

Single venous blood cultures of 8–15 ml from adults and 2–5 ml of venous blood from infants and children were routinely obtained and inoculated into BACTEC plus aerobic bottles (Becton Dickenson). Inoculated BACTEC bottles were incubated at 37 °C in a BACTEC 9240 automated analyser for up to 5 days and sub-cultured when the machine indicated a positive signal. All sub-cultures were plated onto fresh sheep blood agar, MacConkey agar and chocolate agar for bacterial isolation. Plates were incubated at 37 °C in air for 5 days and organisms were subsequently identified by standard methods including API20E and API20NE identification kits (bioMérieux).

### Rectal swab/faecal specimen culture

Stools/rectal swabs were plated onto MacConkey agar to isolate suspected *E. coli* colonies (e.g. lactose-fermenting and non-lactose-fermenting, circular, elevated, entire margins, smooth texture, pink or colourless, dry and shiny). Pooled *E. coli* sweeps on MacConkey agar were inoculated into brain heart infusion (BHI) media supplemented with 20 % glycerol and stored at −80 °C, until plating onto MacConkey agar supplemented with ciprofloxacin (4 mg l^−1^) to select for ciprofloxacin-resistant *E. coli. E. coli* ATCC25922 [ciprofloxacin minimum inhibitory concentration (MIC)=0.008 mg l^−1^] was used as a negative control. All suspected *E. coli* colonies were confirmed using a MALDI MS identification system (Bruker).

### Antimicrobial susceptibility testing

Antimicrobial susceptibility testing was performed by VITEK 2 Compact (bioMérieux) against trimethoprim-sulfamethoxazole, ciprofloxacin, imipenem, meropenem, ceftazidime, cefepime and ceftriaxone. Susceptibility results were interpreted according to the CLSI 2016 guidelines [28]. Detection of ESBL activity was performed for all isolates that were resistant to ceftriaxone using the combination disc method (cefotaxime, 30 µg; ceftazidime, 30 µg; with and without clavulanic acid, 10 µg). ESBL-producing organisms were defined as those exhibiting a >5 mm increase in the size of the zone of inhibition for the beta-lactamase inhibitor combinations in comparison to a third-generation cephalosporin without the beta-lactamase inhibitor.

### Whole genome sequencing

Genomic DNA from *E. coli* ST1193 was extracted using the Wizard Genomic DNA Extraction Kit (Promega) following the manufacturer’s recommendations. One nanogram of genomic DNA from each sample was subjected to library preparation using a Nextera XT kit, followed by whole genome sequencing on an Illumina HiSeq platform (Illumina) to generate 150 bp paired-end reads. Raw sequence data are available in the European Nucleotide Archive (accession numbers in Table S1).

### SNP detection and phylogenetic analysis

Raw Illumina reads were subjected to quality control using FastQC v0.11.5, and FASTX-ToolKit v0.0.14 was used to remove adapters and low-quality bases. Trimmed reads were mapped against the reference genome, MCJCHV-1 (accession number: CP030111), using RedDog pipeline v1.10b (https://github.com/katholt/RedDog). Briefly, RedDog used Bowtie2 v2.2.3 to map all raw reads and SNPs were identified with SAMTools v1.3.1 [29]. High-quality SNPs were then extracted using a standard approach (removing low-confidence alleles with base quality <20 and fewer than five supporting reads) as described previously [30]. Genomic sequences were removed from further analyses if there was evidence suggestive of contamination, i.e. <50 % of reads mapping to the reference genome or the total assembly length being >5.5 Mb [31]. Gubbins v1.4.5 [32] was used to remove recombinant regions from the resulting alignment file and SNPs identified in those regions were subsequently removed, resulting in a final alignment of 759 SNPs. RAxML (Randomized Axelerated Maximum Likelihood) [33] was used to infer a maximum-likelihood (ML) phylogenetic tree using the generalized time-reversible model and a Gamma distribution to model site-specific rate variation (GTR+G). Support for the ML tree was assessed via 100 pseudo-replicates.

To investigate the phylogenetic structure of *E. coli* ST1193 isolates from our study in a global context, we combined genomic data from our ST1193 isolates together with ST1193 genomes downloaded from the Enterobase (http://enterobase.warwick.ac.uk/). Due to the lack of accession numbers and/or metadata (i.e. geographical locations), only 221 ST1193 genomes from the Enterobase were selected for analysis (details in Table S2). SNP calling and analysis were performed as described above, resulting in an alignment of 4564 SNPs. A second ML phylogeny was reconstructed from this SNP alignment, using RAxML with the GTR+G substitution model and 100 bootstrap pseudo-replicates. An *E. coli* ST14cc isolate (accession number: CP012379) was used as an outgroup to root both ML trees. The Interactive Tree of Life (iToL) was used for phylogenetic tree annotation [34].

### Gene content analysis

SRST2 v0.2.0 [35] was used to identify acquired resistance genes, virulence genes, plasmid replicon types, MLSTs and serotypes using the following databases: ARG-ANNOT antimicrobial resistance database [36], BIGSdb virulence genes database (https://bigsdb.web.pasteur.fr), PlasmidFinder [37], *E. coli* MLST scheme (https://pubmlst.org/mlst/) and EcOH serotyping database [38], respectively. SRST2 used Bowtie 2 to map raw reads against the reference databases and SAMtools to identify genes and alleles. All Illumina reads were *de novo* assembled using Unicycler v0.4.8 to generate contigs using the default settings [39]. Comparisons between *bla*_CTX-M-27_-carrying plasmids were performed using blast Ring Image Generator (BRIG) [40].

## Results

### AMR profile, virulence factors and resistome

Among the tested antimicrobials, all 46 *E. coli* ST1193 isolates from this study exhibited resistance to ciprofloxacin. Of these 46 isolates, 32 (70 %) were also ESBL-positive and were resistant to ceftriaxone, ceftazidime and cefepime; one additional isolate was found to produce cephalosporinase and displayed resistance to ceftriaxone and ceftazidime but was susceptibile to cefepime. All isolates were susceptible to meropenem, and all except one were susceptible to imipenem. A high proportion of isolates (83 %, 38/46) showed resistance to co-trimoxazole.

The majority of ST1193 isolates (39/46, 85 %) possessed a K1 capsule and belonged to the O75:H5 serotype; furthermore, 13 % (6/46) belonged to the O-untypable:H5:K-untypable serotype and 2 % (1/46) were of the O18:H5:K-untypable serotype. Overall, most ST1193 isolates carried similar sets of virulence factors, including adhesins (pap/fimH/csgG), toxins (sat/usp/senB), siderophores (fepA/chuA/fyuA/irp1/sitA/iucA/iutA), invasins (ibeB/vat) and haemolysin (hlyE). One isolate carried two additional virulence genes: *cnf* (cytotoxic necrotizing factor) and *hlyA* (haemolysin A) (Table 1). The virulence-associated genes were identified on chromosomal pathogenicity islands (PAIs), plasmids and mobile elements (insertion sequences and transposons). Based on a previously proposed virotype scheme [41], all *E. coli* ST1193 isolates were classified into virotype C, a globally predominant virotype [42] .

**Table 1.** Prevalence of virulence genes in *E. coli* ST1193 (*n*=46) from Vietnam

Virulence gene	Virulence factor	075:H5 (*n*=39)	O-:H5 (*n*=6)	O18:H5 (*n*=1)
**Adhensins**				
*fimH*	Type 1 fimbriae	38 (97 %)	6 (100 %)	1 (100 %)
*papAB*	Pilus associated with pyelonephritis	34 (87 %)	6 (100 %)	1 (100 %)
*csgBCDEFG*	Curli production assembly/transport protein	39 (100 %)	6 (100 %)	1 (100 %)
**Toxins**				
*senB*	Putative enterotoxin	33 (85 %)	5 (83 %)	1 (100 %)
*sat*	Secreted autotransporter toxin	38 (97 %)	6 (100 %)	1 (100 %)
*hlyE/clyA*	Haemolysin	31 (79 %)	6 (100 %)	1 (100 %)
*cnf*	Cytotoxic necrotizing factor	0	1 (16.7 %)	0
*hlyA*	Alpha-haemolysin	0	1 (16.7 %)	0
**Siderophores**				
*fepABCDEG*	TonB-dependent siderophore receptor	39 (100 %)	6 (100 %)	1 (100 %)
*chuA*	Outer membrane haemin receptor	39 (100 %)	6 (100 %)	1 (100 %)
*fyuA*	Outer membrane protein, iron-regulated	39 (100 %)	6 (100 %)	1 (100 %)
*irp1/2*	Yersiniabactin polyketide synthase	39 (100 %)	6 (100 %)	1 (100 %)
*sitABCD*	Periplasmic chelator iron-binding protein	39 (100 %)	6 (100 %)	1 (100 %)
*iucABCD*	Ferric aerobactin receptor	38 (97 %)	6 (100 %)	1 (100 %)
*iutA*	Ferric aerobactin receptor	38 (97 %)	6 (100 %)	1 (100 %)
**Capsula**				
*kpsM/D/T*	Group II capsule	39 (100 %)	6 (100 %)	1 (100 %)
*kpsF*	Group II capsule – K1 group	39 (100 %)	0	0
**Miscellaneous**				
*ibeBC*	Invasion of brain endothelium	39 (100 %)	6 (100 %)	1 (100 %)
*usp*	Uropathogenic-specific protein (bacteriocin)	39 (100 %)	6 (100 %)	1 (100 %)
*vat*	Vacuolating autotransporter toxin	39 (100 %)	6 (100 %)	1 (100 %)

All ST1193 isolates had three amino acid substitutions in the quinolone resistance-determining region, including a single substitution (S80I) in topoisomerase IV (*parC* gene) and two substitutions (D87N, S83L) in DNA gyrase (*gyrA* gene). Sixty-seven per cent of the isolates (31/46) carried a *bla*_CTX-M_ gene and were ESBL-positive. More specifically, 50 % (23/46), 11 % (5/46), and 7 % (3/46) of the isolates harboured *bla*_CTX-M-27_, *bla*_CTX-M-55_ and *bla*_CTX-M-15_, respectively. Furthermore, a majority of *bla*_CTX-M_-carrying isolates had also acquired macrolide and aminoglycoside resistance genes, including *mphA* (29/31, 94 %), *ermB* (15/31, 48 %) and *aac3-IIa/d* (9/31, 29 %). Overall, most isolates carried diverse AMR genes conferring resistance to co-trimoxazole (*dfrA +sulI*/*sulII*/*sulIII*) (36/46, 78 %), streptomycin (*strAB*) (33/46, 72 %), tetracyclines (*tetA/B/M*) (29/46, 63 %), and ampicillins (*bla*_TEM-1-D_, *bla*_OXA-1_, *bla*_DHA-1_ *, bla*_CMY-42_) (31/46, 67 %). Additionally, 2/46 (4 %) isolates carried *catA1*/*catB4* and *qnrB4*, associated with resistance to chloramphenicol and quinolones, respectively.

### Phylogenetic structure of *E. coli* ST1193 isolates from Vietnam

**Fig. 1.**
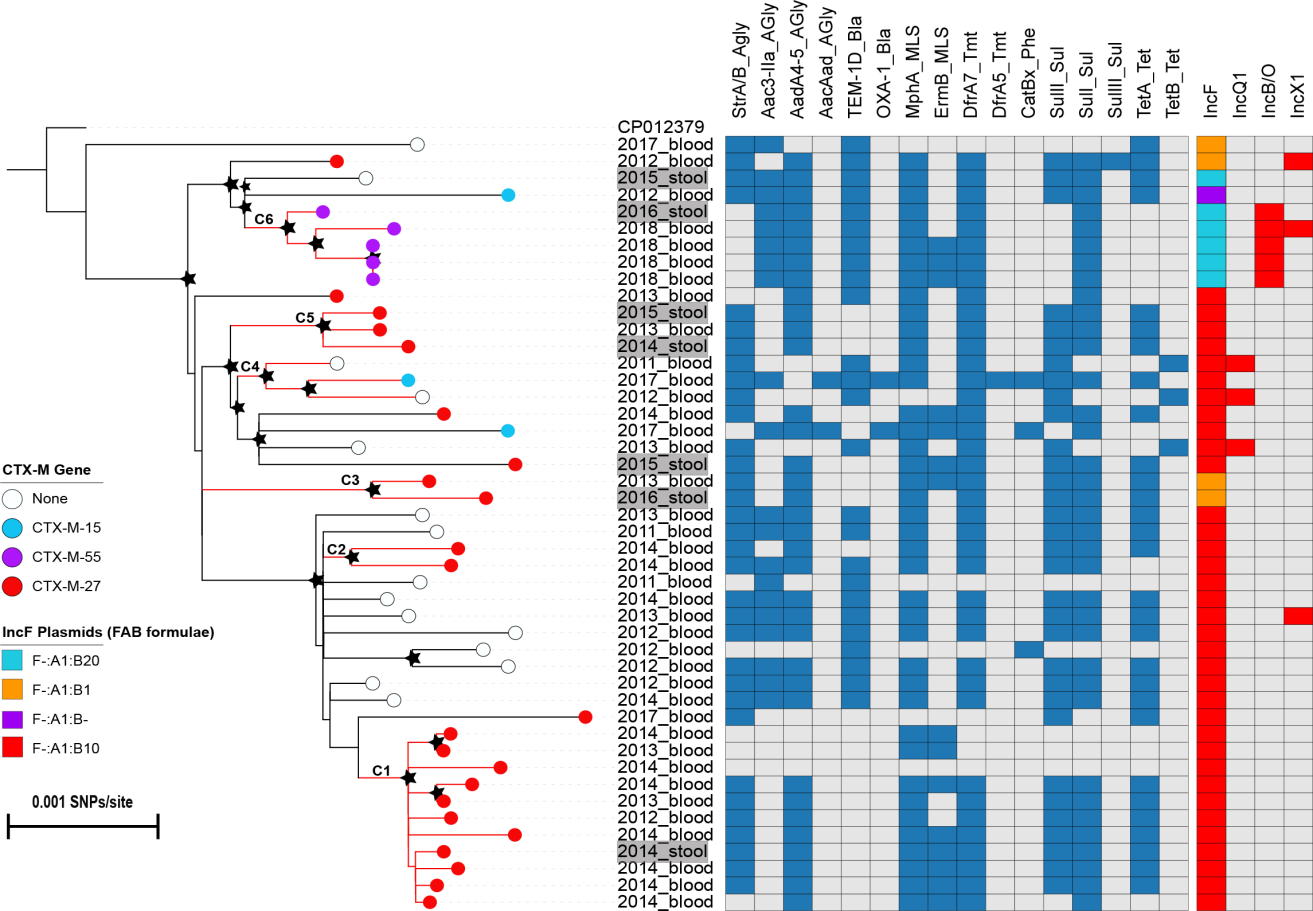
Phylogenetic structure of *E. coli* ST1193 isolates from Vietnam. ML phylogeny of *E. coli* ST1193 in Vietnam. The ML tree was rooted using *E. coli* strain PAR (ST14cc) as an outgroup. Node colours correspond to different variants of the *bla*_CTX-M_ genes. The tip labels highlighted in grey indicate isolates from human carriers. Bar, number of SNPs per site. Black stars indicate bootstrap support values ≥80 % on internal nodes, with larger stars indicating higher bootstrap values. The heat map shows the presence (blue and red colour) or absence (grey colour) of resistance genes and plasmid replicon type/alleles. The multireplicon IncF plasmids are subtyped using FAB (FII:FIA:FIB) formulae by the allele type and number identified for each replicon. Prefixes F- and B- indicate the absence of the FII and FIB replicons, respectively.

We inferred the phylogeny of ST1193 isolates from Vietnam to delineate the pathogen population structure and define the genetic relatedness of ST1193 isolates from patients with BSIs and from human carriers. Although all except one ST1193 isolate fell into a single cluster with a well-supported bootstrap value, we observed discordance between the phylogenetic structure and a number of genotypic (AMR gene profile, plasmid replicon type/allele) and phenotypic (AMR profile, O:H serotype) traits. These data indicate that the local ST1193 population is heterogeneous and dynamic, which suggested that it originated from either a single importation event followed by rapid trait changes, or more likely, multiple importations of distinct ST1193 variants. Based on the bootstrap support values indicating significant clustering, as well as the presence of the *bla*_CTX-M_ gene and the plasmid replicon type/allele, six clusters (C1–C6, cluster sizes: 2–11) are highlighted on the phylogenetic tree (Fig. 1). Four clusters (C1, C2, C3, C5) carried *bla*_CTX-M-27_ and a similar array of AMR genes: *strAB-aadA5-mphA-ermB*(+/−)*-dfrA7-sulI-sulII-tetA.* These AMR genes were located on an IncF-:A1:B10 (C1, C2, C5) or an IncF-:A1:B1 plasmid (C3). One additional cluster (C6) harboured *bla*_CTX-M-55_ on an IncB/O plasmid and a dissimilar array of AMR genes: *aac3IIa-aadA5-bla_TEM-1D_-mphA-ermB(+*/*−*)*-dfrA7-sulI* on an IncF-:A1:B20 plasmid. The C6 cluster contained one isolate from a human carrier and four isolates from neonatal sepsis cases. Notably, we found a cluster (C4) comprising three isolates, two of which lacked a *bla*_CTX-M_ gene (identified in 2011 and 2012), while the remaining one (identified in 2019) carried an AMR gene array, *bla*_CTX-M-15_-*aac3IIa-bla*_OXA-1_ *-dfrA5-catBx-sulI-tetA.* These data suggest that, in at least some lineages, resistance within the ST1193 population developed during circulation in Vietnam.

Among the identified clusters, there were four clusters (C1, C3, C5, C6) within which the ST1193 isolates from BSIs and human carriers clustered tightly together and exhibited the same AMR gene profiles, plasmid IncF alleles and O:H serotypes. These findings suggest that ExPEC ST1193 is probably able to establish asymptomatic carriage in the human intestine, which may have facilitated its transmission in the population.

### Phylogenetics of Vietnamese ST1193 isolates in a global context

**Fig. 2.**
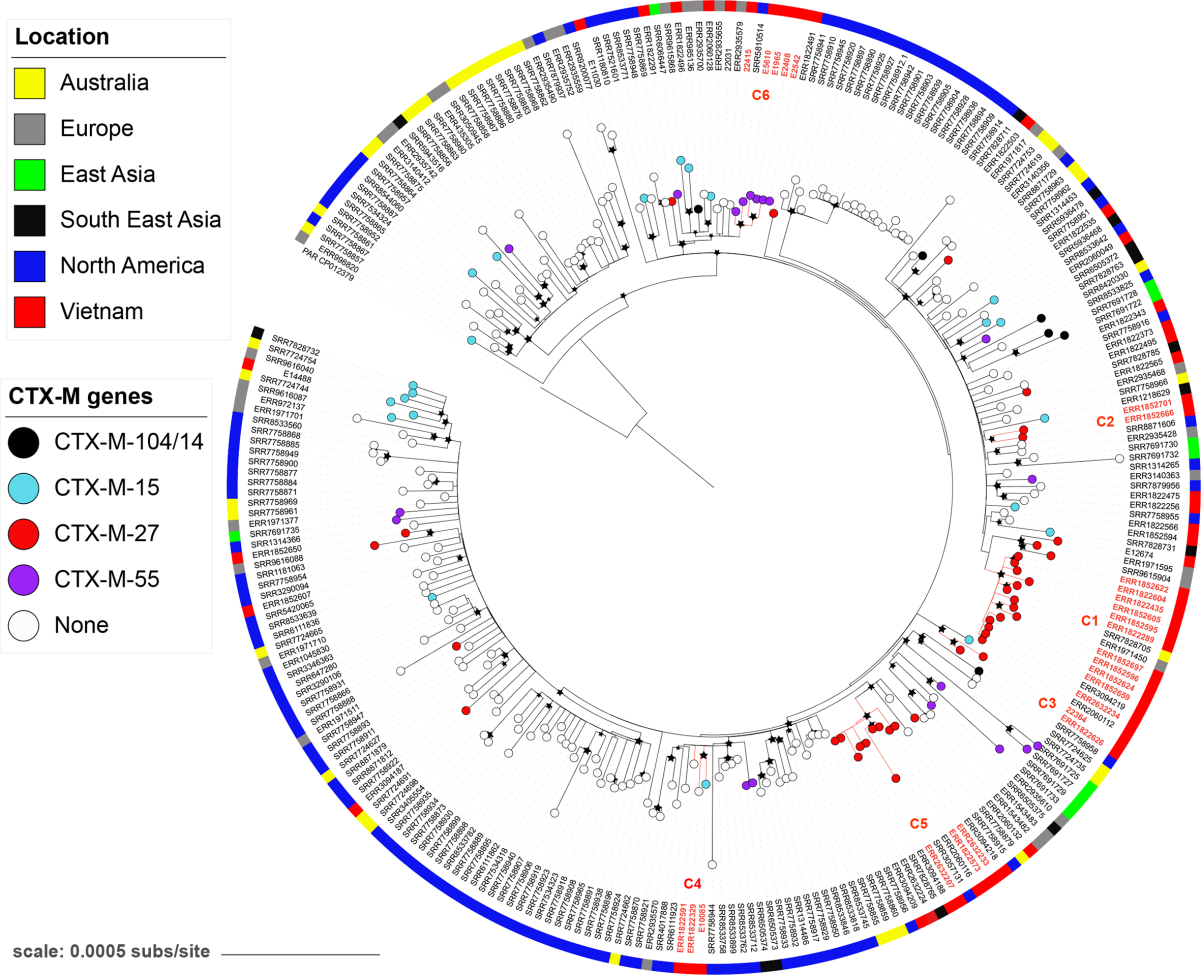
Phylogeny of Vietnamese *E. coli* ST1193 isolates in a global context. ML phylogeny reconstructed from a global database of ST1193 *E. coli,* rooted using *E. coli* strain PAR (ST14cc) as an outgroup. The tip colours correspond to different variants of the *bla*_CTX-M_ gene. The red tip labels indicate isolates within the six clusters C1–C6 identified in this study. Bar, number of SNPs per site. Black stars indicate bootstrap support values ≥80 % on internal nodes, with larger stars showing higher bootstrap values. The outer ring shows the location from which the isolates were reported.

To provide further insight into the population structure and transmission dynamics of ST1193 isolates in Vietnam in a broader context, we inferred a phylogeny that included our isolates together with a global collection of ST1193 from the Enterobase. Overall, we observed that the ST1193 isolates from Vietnam were dispersed across the global phylogenetic tree. Furthermore, the six identified clusters from Vietnam (C1–C6) did not share a single recent common ancestor; instead, these clusters were probably associated with multiple independent introduction events (Fig. 2). We also found that our ST1193 isolates clustered together with isolates obtained from the Enterobase on numerous occasions, including those collected in Vietnam [43], Australia [23], the USA [44], Denmark [45] and Singapore [46]. These findings reveal that multiple importations of genetically and/or phenotypically dissimilar ST1193 variants in combination with local evolution via AMR gene/plasmid acquisitions were likely to be the primary factors shaping the population structure and AMR profiles of ST1193 in Vietnam.

### Comparative analysis of *bla*_CTX-M-27_-carrying plasmids in ST1193 isolates

**Fig. 3.**
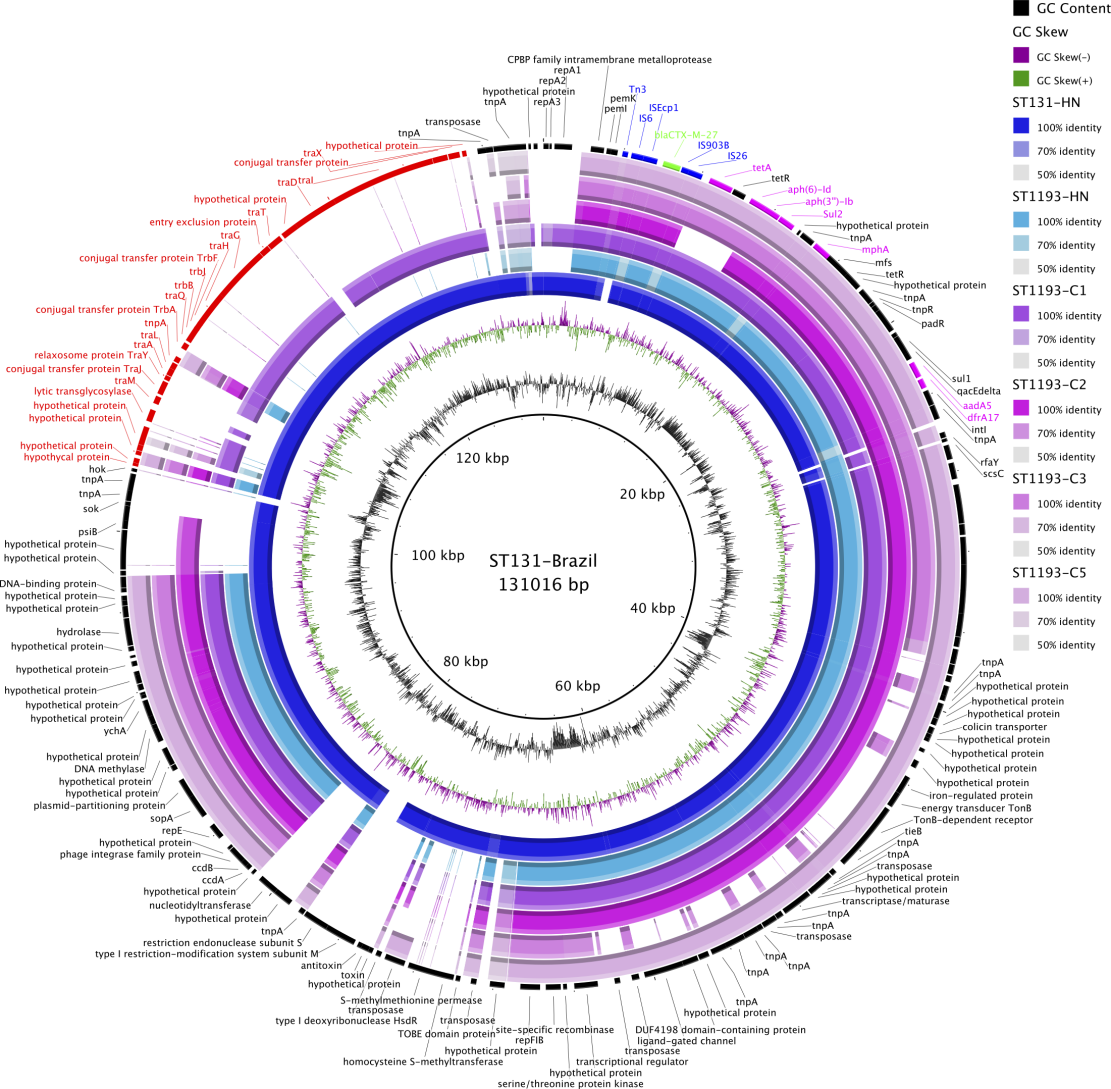
blast comparisons of *bla*_CTX-M-27_-carrrying plasmids identified in our ST1193 isolates and previously published ST1193 and ST131 isolates from Vietnam. The central circle indicates the full reference sequence of *bla*_CTX-M-27_-carrying plasmid pMO (IncF1:A2:B20) from an ST131 isolate in Brazil (accession number: MG886288). The two innermost rings show the GC content and GC skew of the reference plasmid. The remaining concentric rings show the nucleotide similarity between the reference plasmid and the *bla*_CTX-M-27_-carrrying plasmids identified in previously published ST131 and ST1193 isolates from Vietnam (isolates: ST131-HN, ST1193-HN; accession numbers: SRR11392822, SRR11392956) and our ST1193 isolates (**C1, C2, C3, C5**). The outermost ring shows the annotations of the reference plasmid, in which the conjugation module shared between IncF-:A1:B10 (**C1**) and IncF1:A2:B20 plasmids is highlighted in red.

Here, we found that *bla*_CTX-M-27_ was the most common *bla*_CTX-M_ variant identified in ST1193 isolates. Recently, the emergence of *bla*_CTX-M-27_ has also been documented in a potential epidemic subclade (C1-M27) of ExPEC ST131, associated with a large transferable IncF1:A2:B20 plasmid of 131 kb [47, 48]. We sought to compare the genetic structures of *bla*_CTX-M-27_-carrying plasmids in our ST1193 isolates (C1, C2, C5: IncF-:A1:B10; C3: IncF-:A1:B1) with representative *bla*_CTX-M-27_-carrying plasmids published previously in ST1193 (IncF-:A1:B10) and ST131 (IncF1:A2:B20) isolates from Vietnam [22]. Our analysis showed that the IncF-:A1:B10 plasmids in ST1193 isolates shared a similar genetic backbone (coverage: 63.3–82.8 %, identity: 99.5–99.8 %) with IncF1:A2:B20 plasmids in ST131 isolates. Notably, the IncF-:A1:B10 plasmid found within ST1193 cluster C1 also carried the conjugation module similar to the IncF1:A2:B20 plasmid in ST131, indicating that this plasmid was self-transferable (Fig. 3).

## Discussion

In this study, we present the first genomic insight into the emerging pandemic fluoroquinolone-resistant ST1193 clone in Vietnam. Our study uncovers the endemic circulation of a heterogeneous ST1193 population in this setting, predominantly driven by multiple strain importations, many of which are associated with resistance to third-generation cephalosporins. Our limited data also show that imported strains can further acquire AMR genes from the local gene pool following introduction to a new population; we speculate that this may be due to bacterial adaptation in response to heavy antibiotic usage in this setting. Our findings raise concerns that new drug-resistant ST1193 variants can emerge locally when they enter new human populations with enriched AMR reservoirs and high antimicrobial selection pressures, similar to what was observed during the introduction and microevolution of ciprofloxacin-resistant *Shigella sonnei* in Vietnam [49]. Here, we found a dominance of the *bla*_CTX-M-27_ ESBL gene in ST1193 isolates and a high degree of synteny in the backbone of *bla*_CTX-M-27_-carrying plasmids in ST1193 and ST131. Our findings suggest that *bla*_CTX-M-27_-carrying plasmids have either been transferred between ExPEC clones or independently acquired from the same reservoir; additionally, the plasmid acquisitions appear to be associated with epidemiological success of ExPEC strains. The reservoirs and factors driving the emergence of *bla*_CTX-M-27_ in ST1193 and other ExPEC clones warrant further investigations. We advocate for the integration of molecular and genomic typing into routine surveillance of ExPEC in low- and middle-income countries to closely monitor its transmission and to rapidly identify novel drug-resistant variants of ExPEC lineages, as a means of informing healthcare providers of their presence and improving public health and infection control measures.

To date, the factors contributing to the epidemiological success of ExPEC clones are not well understood [17]. It has been postulated that virulence and AMR gene acquisitions are not key determinants of their clonal expansion, while contaminated food products may play an important role in the global dissemination of ExPEC pandemic lineages [16]. However, the potential reservoirs for ExPEC are notoriously diverse, and include the human intestinal tract, companion animals, food animals, retail meat products and environmental sources; additionally, various ExPEC lineages may be associated with distinct host ranges and primary transmission routes [50–54]. In this study, we found that ST1193 isolates from BSI cases and human carriers from within the same population are genetically closely related, indicating that this clonal group can potentially colonize the healthy human gut and transmit from human to human.

Previous studies involving genetic characterization of *E. coli* from humans and animals in Vietnam have reported the absence of ST1193 from farm animals [55–57]; notably, in one chicken farm, ST1193 isolates were recovered from two farmers but were absent in chicken samples [55]. Furthermore, the presence of phylogenetic group B2 *E. coli* in pre-processed animal-based food products in HCMC is reportedly very low (6/342 isolates) [58]. This absence of *E. coli* ST1193 in animals and animal-based food products suggests that animal-to-human transmission is probably not a major route of transmission in this setting; thus, we speculate that ST1193 strains primarily transmit between humans. This also means that the human gut may act as an important reservoir for the maintenance, transmission and evolution of drug resistance in this clone. Previous studies show that social interaction and local/international travel can facilitate the rapid transmission of drug-resistant bacteria [50, 59, 60]. Here, we observe a high level of geographical mixing of genetically linked ST1193 organisms over limited time scales, indicating rapid transmission of this clone across the globe, probably through international travel or the consumption of import/export food products. The low frequency of *E. coli* ST1193 in food animals and food products to date implies that the latter of these explanations is less likely, although large-scale One Health genomic approaches are needed to generate conclusive evidence regarding its transmission pathways and the major vehicles of transmission.

The study has some limitations. The number of ST1193 isolates from human carriers was small and restricted to children less than 5 years of age at a single time point. Therefore, there is limited understanding of the carriage rate and duration of colonization of ST1193 in different age groups. These data are necessary to determine the fraction of the human population who are likely to carry and contribute to the spread of these organisms. Furthermore, the sampling of ST1193 isolates from bloodstream infections was intermittent and involved different age groups (neonates, adults), and thus our inference about the genetic and phenotypic traits of the imported strains was limited. For example, we were largely unable to determine whether the AMR gene/plasmid acquisitions occurred before or after importation, or to track the continued evolution and transmission of the *bla*_CTX-M_-carrying ST1193 clusters. Despite these limitations, our study represents the first molecular description of the ExPEC ST1193 clone in Vietnam and underlines the potential role of human carriers as a vehicle of pathogen transmission for further investigations.

In this study, we report the endemic circulation of a highly dynamic population of ExPEC ST1193 in humans in Vietnam, largely driven by multiple strain importations. ST1193 isolates in Vietnam display a high level of resistance to multiple antimicrobial classes including fluoroquinolones, cephalosporins, macrolides and aminoglycosides. These organisms have the potential to colonize healthy individuals, induce BSIs and acquire additional resistance genes while circulating in this setting. The observed rapid global distribution of *E. coli* ST1193, along with its MDR phenotype and the shortage of new antimicrobial therapeutics, emphasizes the urgent need to control the spread of this pathogen. We call for local and international AMR genomic surveillance across sectors (i.e. clinical, veterinary, agricultural and environmental) to capture new emerging ExPEC clones and to gain deeper understanding of the origins and transmission patterns of these pathogens.

## Supplementary Data

Supplementary material 1

Supplementary material 2
